# The Effect of Immediate Post-Training Active and Passive Recovery Interventions on Anaerobic Performance and Lower Limb Flexibility in Professional Soccer Players

**DOI:** 10.2478/v10078-012-0013-9

**Published:** 2012-04-03

**Authors:** Ezequiel Rey, Carlos Lago-Peñas, Luis Casáis, Joaquín Lago-Ballesteros

**Affiliations:** 1 Faculty of Education and Sports Sciences, University of Vigo. Pontevedra, Spain.

**Keywords:** soccer, recovery, cool-down, fatigue

## Abstract

The capacity to recover from intense training, competition and matches is considered an important determinant in soccer performance. At present, there is no consensus on the effect of post-training recovery interventions on subsequent training session. The aim of this study was to determine the effectiveness of active (12 min submaximal running and 8 min of static stretching) and passive recovery (20 min sitting on a bench) interventions performed immediately after a training session on anaerobic performances (CMJ, 20 m sprint and Balsom agility test) and lower limb flexibility 24 h after the training. During two experimental sessions, 31 professional soccer players participated in a randomized fully controlled trial design. The first session was designed to evaluate the player’s anaerobic performances and lower limb flexibility (pretest). After baseline measurements, participants performed a standardized soccer training during which heart rate and RPE were recorded to evaluate the training load. At the end of the training unit all players were randomly assigned to the active recovery group and the passive recovery group. A second experimental session was organized to obtain the posttest values. Players performed the same test, administered in the same order than in the first trial. No significant differences between groups were observed in heart rate and RPE. No significant effect due to recovery interventions was found on lower limb flexibility and anaerobic performances except CMJ that posttest value was significantly greater in the active recovery group than in the passive group (p < 0.05).

## Introduction

Soccer players are often exposed to demanding training and competition schedules, which may include repeated, high-intensity exercise sessions performed on consecutive days, multiple times per week (King and Duffield, 2009). Each training and game places high physical demands on players as they experience repeated moderate and rapid accelerations and decelerations, explosive jumps, and muscle damage from eccentric loading or contact trauma (Reilly et al., 2008). Excessive volumes of intense training and competition, particularly with minimal recovery time, can place great physiological demands on the musculoskeletal, nervous, immune, and metabolic systems, potentially causing a negative effect on subsequent exercise performance (Reilly and Ekblom, 2005), and predispose some players to overload injuries (Barnett, 2006), especially during a congested fixture period where players are required to compete and train repeatedly within a short time frame (Dupont et al., 2010; Rey et al., 2010). Therefore, the capacity to recover from intense training and competition is considered an important determinant of subsequent performance (Odetoyinbo et al., 2009).

To facilitate the recovery process, different post-exercise recovery modes have been suggested, broadly classified into two categories (Bompa, 1999): (1) active recovery or (2) passive recovery. Active recovery may include jogging or submaximal running and stretching exercises. In practice, these popular and current active recovery strategies are used for the purposes of enhancing recovery during cool down protocols both after training and after matches in professional soccer clubs (Dabedo et al., 2004). However, there is insufficient scientific evidence on the mechanisms and outcomes available that support their implementation (Barnett, 2006). The theoretical overall advantage reported that submaximal running establishes a greater blood flow to muscles, prevents venous pooling in the muscles after exercise, facilitates restoration from metabolic perturbations, attenuates the induction of muscle soreness, and increases muscle-damage recovery (Baldari et al., 2004; Tessitore et al., 2007). Static stretching after exercise is commended as a preventative measure for delayed-onset muscle soreness and improved range on motion through dispersion of oedema or tension reduction of the muscle-tendon unit (Montgomery et al., 2008).

To our knowledge, there are only two scientific reports evaluating the effects of active recovery in male soccer players (Reilly and Rigby, 2002; Tessitore et al., 2007). Reilly and Rigby (2002) examined the efficacy of an active cool-down in two groups of amateur soccer players. One group did an active cool-down after a first match and a controlled recovery regime the week after before completing a second game in which no formal cool-down was conducted. The procedure was reversed in a second group to balance the order of administering the experimental cool-down. The active cool-down consisted of three phases: (1) 5 min of jogging, (2) 5 min of stretching and (3) a further 2 min lying prone with legs raised and “shaken down” by another player. The results showed that players who did active recovery immediately after a game had lower muscle soreness ratings and were closer to their pre-match vertical jump and short running performance measures two days after a game than when no recovery was undertaken. The trends in the data indicated that players cooling down after a midweek match could be adequately recovered for a weekend game 72 h later. On the other hand, Tessitore et al. (2007) examined the efficacy of recovery interventions in maintaining anaerobic performances and subjective ratings before starting the afternoon training session in young elite soccer players. Although no main effect of recovery intervention was observed on anaerobic performances, active recovery and electrostimulation were more beneficial than water aerobic exercises and passive recovery for reducing muscle pain. In both studies the sample size was relatively small (n = 14 and 12, respectively).

The inconclusive findings and the relatively small sample size of preceding investigations suggest that further research is needed to solve the ambiguity of the relation between recovery interventions, athletic performance and physical and physiological parameters. Therefore, the aim of this study was to determine the effectiveness, if any, of active and passive recovery interventions performed immediately after a training session on anaerobic performances and lower limb flexibility 24 h after the training. On the basis of the theory that light muscle activity may accelerate the return of homeostasis in exercised muscle, it was hypothesized that active recovery modality would be more effective than passive recovery in professional soccer players.

## Material and Methods

### 

#### Experimental Design

**Figure 1:**
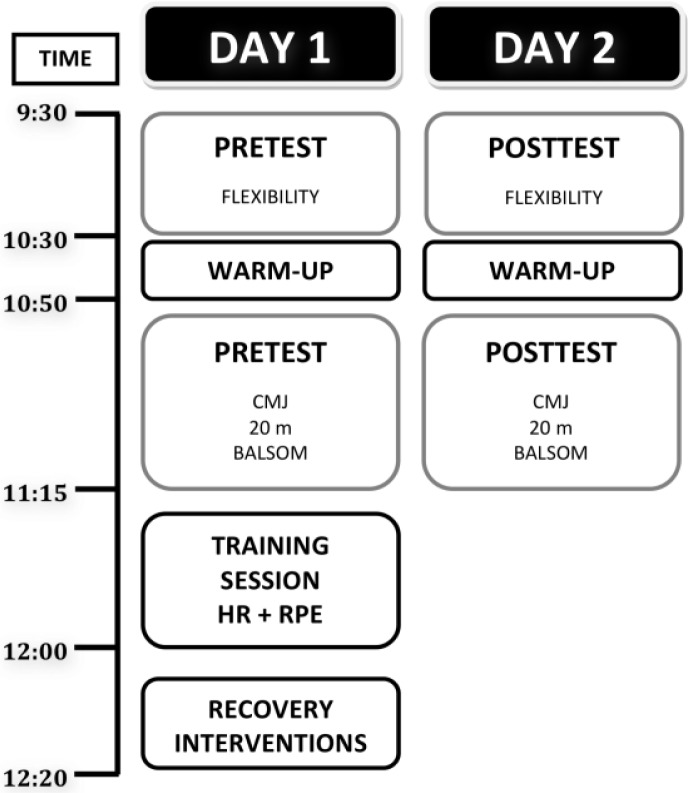
The experimental design. CMJ= countermovement jump; HR= heart rate; RPE= rating of perceived exertion

Despite the popularity of the implementation of these aforementioned recovery strategies in team sports, there is limited evidence to suggest the effectiveness of these methods on subsequent athletic performance. Therefore, a randomized fully controlled trial design, including two experimental sessions (Figure 1), was used to determine the effect of 2 post-training recovery modalities on physical performance 24 h after a training session, designed to stimulate the demands of a soccer game. It was considered that examining elite soccer players during their actual training period would increase the relevance and the applicability of the results. The 2 modalities were as follows: (a) active recovery (ACT), and (b) passive recovery (PASS). Participants were required to wear the same athletic equipment and measurements were conducted at the same time of the day to minimize the effect of diurnal variations on the selected parameters during the two experimental sessions.

#### Sample

Thirty-one professional soccer players (age: 23.5 ± 3.4 years; body height: 179.9 ± 5.1 cm; body mass: 75.7 ± 4.2 kg) participated in this study. All participants were provided with written and verbal information on the objectives of the study, and they completed an informed consent document. This investigation has been performed in accordance with the ethical standards in sport and exercise science research (Ryan et al., 2009). To avoid the influence of different player’s fitness and skill levels on the effects of the recovery interventions, participants were selected on the basis on their participation in Spanish Soccer. Typical training of the players compromised 5–7 full team practices (90–120 min each) for a total training load of approximately 8 – 10 h per week, thus providing adequate specific preparation. Because the physical loading of goalkeepers differs from that of field players, they were not included in the study. The physical characteristics of the players are shown in Table 1.

#### Experimental Procedures

Two consecutive experimental sessions were organized during the in-season soccer training period. The testing sessions were conducted in an outdoor training field at a temperature of 21 ± 4º C and humidity of 73 ± 8 %. Participants were required to present in a rested state at the same time each morning during the 2 experimental sessions (9:30 h). The first session was designed to collect the player’s physical measurements (pretest), consisting of 2 data collection stages (Figure 1) at 9:30 h (flexibility test) and 10:50 (anaerobic evaluation), preceded by a 20 min active warm-up. Immediately after, all participants performed a standard soccer training consisting of a 45 min program, including a 15 min maximal intensity intermittent exercise (20 x 30 m, with a 30 s rest period between each sprint) (Balsom, 1994) and a 30 min group specific aerobic endurance drill (4 x 4 min of 5 a side game, including goalkeepers, in an area of 40 x 50 m, with a 3 min active rest at 65 % of maximal aerobic velocity between sets) (Hoff et al., 2002). To ensure that the training load did not vary between experimental and control groups, the player’s heart rate (HR) was recorded during the entire training unit (Polar Team System, Polar Electro, Kempele, Finland) and at the end of the session they were asked to provide a rating of perceived exertion (RPE) on a 15 point scale (Borg, 1998), ranging from “light” (6 points) to “maximal effort” (20 points). At the end of the training unit all players were randomly assigned to the active recovery group (n = 15) and the passive recovery group (n = 16). A second experimental session was organized to obtain the posttest values. Players performed the same test, administered in the same order than in the first trial.

*Recovery modalities.* The recovery protocols to be performed at the end of the first experimental training session were as follows: active recovery (ACT) consisted of 20 min low-intensity exercises, including 12 min submaximal running at 65% of MAV [2] and 8 min of static stretching, involving 3 bilateral repeats of 30 s held stretches to the hamstring, quadriceps, gastrocnemius, and adductor muscles (Ryan et al., 2009); passive recovery (PASS) during which the players sat on a bench, lasted 20 min, according to the duration of the active recovery protocol. The players were instructed not to use other forms of recovery (i.e., massage, cold water immersion, etc.) during the two experimental sessions.

#### Anaerobic Evaluations

Vertical jump, sprint velocity and agility are considered as determining factors of professional soccer players (Stølen et al., 2005). These indicators were used in this study as simple and reliable measures of anaerobic performance. Before the anaerobic test the players performed a 15 min structured warm-up adapted from Olsen et al. (2005) to prevent lower limb injuries, during which they carried out transit mobility, technique, balance, and power exercises. After the warm-up, the players performed 3 tests, administrated in the same order throughout the study: (a) countermovement jump (CMJ); (b) 20 m sprint; and (c) Balsom agility test (Balsom, 1994). Participants were habituated to these tests, routinely administrated during the soccer season. For each test, players were allowed 2 trials, with a 3 min recovery period between. The best trial was used for subsequent analysis (CV < 0.5).

**Figure 2:**
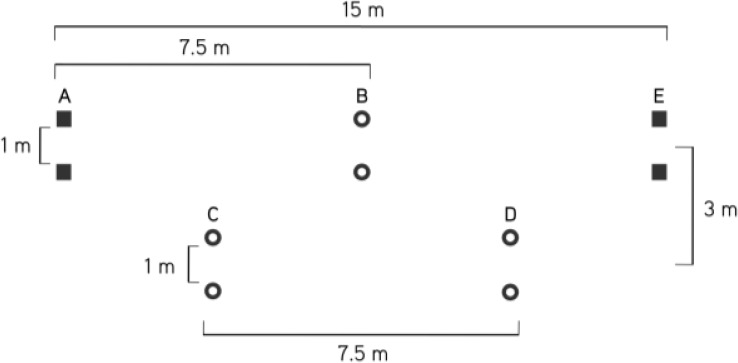
Balsom agility test course. Players start at point A, and sprint to the cones at point B. They turn at point B, sprint back through point A, turn to the left and sprint through point C to point D. They turn at point D and then sprint back through C, turn to the right and sprint through point B to the finishing gate shown at point E. All distances are indicated on the diagram

CMJ was performed on a force plate (Ergo Jump Bosco System, Globus, Treviso, Italy), which calculates the height of the jump. For the CMJ, from standing position with the hands fixed on the hips, the soccer players were required to bend their knees to a freely chosen angle and perform a maximal vertical thrust (Rodacki et al., 2002). Participants were instructed to keep their body vertical throughout the jump, and to land with knees fully extended. Any jump that was perceived to deviate from the required instructions was repeated. The 20 m sprint and Balsom agility tests were measured by means of a dual infrared reflex photoelectric cell system (Heder, HL2-11). Players began from a standing start, with the front foot 1 m from the first timing gate. During the two experimental sessions the participants were required to wear the same shoes to avoid the effects of different athletic equipment on anaerobic performances. Figure 2 shows the Balsom agility test course.

#### Flexibility Measurements

Flexibility of the hamstring, quadriceps, adductor, and gastrocnemius muscles was measured goniometrically on the dominant leg. The dominant leg was defined as the preferred kicking leg. The same two collaborators, with a degree in Sports Science, performed all measurements. As reported by Witvrouw et al. (2003) previous research has indicated that the goniometric measurements are reliable.

#### Diet Control and Fluid Intake

In the early of two experimental sessions, subjects were provided with individual 250 ml water bottles and were encouraged to drink ad libitum before, during, and after the training. The players were instructed to drink only from their own bottles. The food intake was standardized for all players during the whole study period. To diet control each participant was given a meal plan composed by a nutritionist (Reilly and Ekblom, 2005).

### Statistical Analysis

Data are presented as means ± SD. A 0.05 level of confidence was selected throughout the study. Statistical analyses were conducted using the statistical package SPSS for Macintosh (version 18.0, Chicago, IL, USA). To evaluate the stability of the training load between groups (ACT vs. PASS) according to the HR and RPE values independent-samples *t*-test and *Mann Whitney U* test were used, respectively.

To study the effectiveness of different postgame recovery interventions, the independent variable was the type of recovery (ACT and PASS) and dependent variables were the anaerobic (CMJ, 20 m sprint, and Balsom agility test) and flexibility tests. A MANOVA with testing time (pre-post) as within-factor and recovery modes (ACT, PASS) as between-factor, using absolute values, was applied to anaerobic and flexibility performances. Furthermore, to provide meaningful analysis for comparisons from small groups, the Cohen’s effect sizes (ES) were also calculated. An ES ≤ 0.2 was considered trivial, from 0.3 to 0.6 small, < 1.2 moderate and > 1.2 large (Hopkins and Batterham, 2006).

## Results

### Stability of Training Load

**Figure 3:**
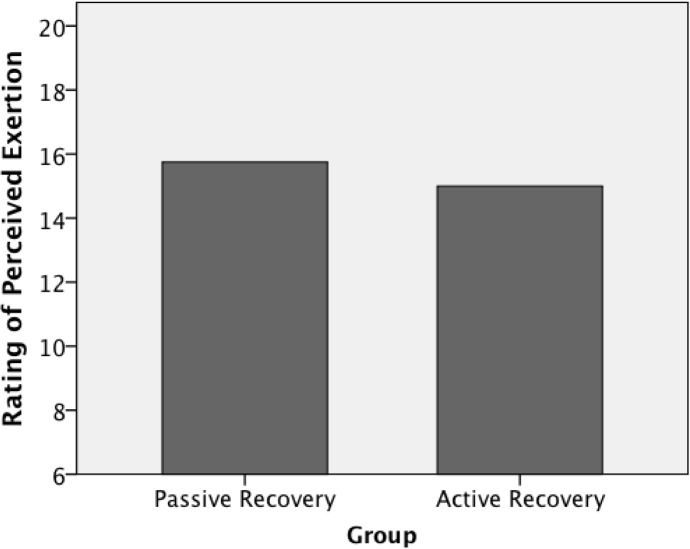
Intensity of soccer training for passive and active recovery groups. Means of RPE

There were no significant differences in RPE (Figure 3) and HR between groups during training session. The average HR for PASS and ACT was similar (166 ± 6 and 164 ± 6 b x min^−1^, respectively, p > 0.05). Altogether, the levels of RPE and HR values indicate the relative high intensity of the training and the homogeneity of training load between active and passive group.

#### Effects of Recovery Interventions

At baseline, there were no significant differences in absolute values for CMJ, 20 m sprint, the Balsom agility test, and flexibility between active and passive recovery groups. No significant effect due to recovery interventions was found on anaerobic performances except the CMJ in which the posttest value was significantly greater in the active recovery group than in the passive group (p < 0.05).

**Figure 4:**
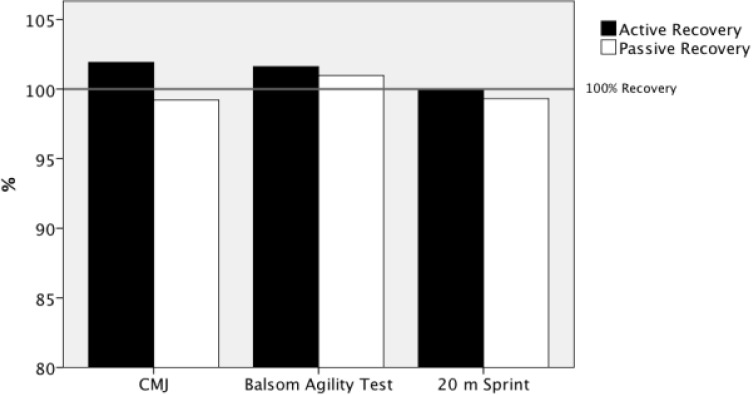
Percentages of variations of anaerobic test performances for passive and active recovery groups. Mean recovery approached 100% for each recovery intervention

Table 2 reports the performance scores for the variables measured during pretest and posttest stages. Mean recovery approached 100% for each intervention (Figure 4), with better results for the active recovery group. Generally, trivial to moderate effect sizes were found (range, 0.01–0.94).

Means and standard deviations for the flexibility measurements are presented in Table 3. For both groups, posttest values showed a lower mean flexibility in quadriceps muscle (p < 0.05).

At pretest, there was a significant difference in absolute values for hamstring muscle flexibility between active and passive recovery groups (p < 0.05). No significant effect due to recovery interventions was found on lower limb flexibility. Trivial to moderate effect sizes were found (−0.15, −0.94).

## Discussion

Several postgame recovery interventions have been suggested to enhance performance, and to avoid the incidence of muscle damage, the symptoms of overreaching, and the lower limb injuries that result from the high frequency and intensity of training, despite the lack of scientific agreement regarding their efficacy (Barnett, 2006; Reilly and Ekblom, 2005; Robson-Ansley et al., 2009). Thus, the present study was mainly designed to investigate the effects of immediate post-training active (low intensity running and static stretching, mostly used or recommended in soccer) and passive recovery interventions on anaerobic performances and lower limb flexibility in professional soccer players.

The main findings of this study were: (a) active recovery induced significant differences in CMJ performance 24 h after training session; (b) active recovery, after specific soccer training, did not have a positive effect on 20 m sprint and Balsom agility test performances compared with passive recovery modality; (c) no significant differences were recorded between recovery conditions on preferred lower leg flexibility in the posttest results. However, this study has 3 potential limitations. The first is that the observation period (two experimental sessions) might be too short to evaluate the effect of recovery interventions over time. A longer period is needed (for example one week) to analyze the effectiveness of the two types of rest in detail. However, it is unfeasible to hypothesize that coaches and professional players will be available for a longer experimental study, which could interfere with their training program. The second limitation is that other recovery indicators such as biochemical parameters were not included in the study in an attempt to keep it simple, noninvasive, and practical. Third is the use of a single, practical, short-term physical measure to assess performance change. Consequently, it is not known how the different recovery methods impact on longer duration physical performance such as intermittent running (Kinugasa and Kilding, 2009).

In this study a standard training session was administrated and the player’s HR and RPE were used to monitor their training intensity. The lack of significant differences between experimental and control group for both HR and RPE confirms that the same training load was administrated. The HR was in agreement to that reported previously for an intense training workload in professional soccer players (Hoff et al., 2002). The participants perceived the intensity of the training as hard, similar to that observed by Impellizzeri et al. (2004).

In the present study the 20 m sprint and the Balsom agility test did not show substantial differences among the two experimental conditions compared with baseline (before training) measurements. This finding agrees with previous studies in Australian football players (Dawson et al., 2005) and young soccer players (Tessitore et al., 2007). Collectively, this might suggest that regardless of the nature of physical measure, the short-term effect of different recovery strategies on physical performance would seem to be not performance enhancing but to maintain performance. Another possible explanation might be the fact that the studied variables were not sensitive enough to address changes in the recovery process or that other recovery interventions might be more effective. Nevertheless, significantly better mean CMJ performance was 24 h following active recovery, indicating that recovery mode could represent valuable aids for muscle recovery function. These findings are generally the same as reported by Reilly and Rigby (2002) in amateur soccer players, who found that their 12 min active recovery immediately after a soccer match left players in a better functional state (closer to their pre-match jump scores) 24 h later than if they had done nothing. Conversely, the results of present study disagree with other investigations in young soccer players (Kinugasa and Kilding, 2009; Tessitore et al., 2007), and in elite female soccer players (Andersson et al., 2008), possibly due to differences such as the sample size and duration of active recovery protocol observed among studies. Taken as a whole, the results of anaerobic performance suggest that players after active recovery intervention may be able to produce efforts that are equal or close to their maximum. It is also possible that aerobic or repeated effort performance may have been facilitated by the recovery procedures, and this should be explored in further research (Dawson et al., 2005).

Flexibility scores were similar to those reported by Witvrouw et al. (2003) in male professional soccer players. Small deteriorations from the first to second experimental session occurred in all muscle groups. However, no significant differences were observed between recovery procedures. These results are similar to those reported by Dawson et al. (2005) and Montgomery et al. (2008) in Australian football and basketball players, respectively. Both studies used the sit-and-reach test to evaluate whole body flexibility. Based on these results, it may be suggested that following a specific training session, the acute effects of static stretching might not be sufficient to reduce the muscle tightness and increase the range of motion 24 h after.

Recovery methods during cool-down should be viewed as an integral part of training sessions and should be conducted based on several criteria such as the fatigue levels of players according to the training load and time required to recover. Consequently, further studies are necessary to address and determine the optimum quantity and quality of exercise during active recovery period depending of the training characteristics. Coaches also should give consideration to appropriate diet, rehydration, and an adequate passive rest and sufficient sleep (Robson-Ansley et al., 2009). Surely, the underlying mechanisms of recovery following soccer training in professional players remain debatable and further studies are necessary. However, the data reveal positive effects of active recovery intervention on CMJ performance, thus enhancing the player’s anaerobic working capacity toward the next training.

In conclusion, the results of the current study indicate that post-training active recovery intervention may help in restoring CMJ performance but do not represent performance enhancements in the 20 m sprint, Balsom agility test and lower limb flexibility in professional soccer players. It is possible to hypothesize that longitudinal research protocols could be more successful in providing valuable information for the coach on the effectiveness of recovery interventions. Therefore, further studies on post-training recovery modalities that maintain ecological settings are strongly recommended.

## Figures and Tables

**Table 1:** Physical characteristics of the players participating in the study

**Group**	**N**	**Age (yr ± sx)**	**Body mass (kg ± sx)**	**Body Height (cm ± sx)**
Passive Recovery	16	23.3 ± 3.3	75.1 ± 3.1	179.7 ± 5.4
Active Recovery	15	23.6 ± 3.5	76.2 ± 5.2	180.1 ± 5.7

**Table 2:** CMJ height, 20 m sprint time, and Balsom agility test time for the active recovery group and passive group at baseline and 24 h after the training session

	***N***	**Pretest (Baseline)**	**Posttest (24 h after training)**
**CMJ (cm)**			
Active Recovery	15	41.3 ± 4.4^*^ (0.21)	42.6 ± 3.9^#^ (0.94)
Passive Recovery	16	40.5 ± 3.1	39.2 ± 3.3
**Balsom Agility Test (s)**			
Active Recovery	15	11.15 ± 0.57 (0.03)	11.03 ± 0.69 (−0.09)
Passive Recovery	16	11.13 ± 0.58	11.09 ± 0.61
**20 m sprint (s)**			
Active Recovery	15	3.12 ± 0.11 (0.19)	3.12 ± 0.11 (0.01)
Passive Recovery	16	3.10 ± 0.09	3.12 ± 0.08

* Significant differences (p < 0.05) with posttest.

# Significant differences (p < 0.05) with passive recovery group.

ES (with respect to passive recovery group values) are shown in parentheses

**Table 3:** Means and standard deviations of quadriceps, hamstring, adductor, and gastrocnemius muscle flexibility (expressed in degrees)

	***N***	**Pretest (Baseline)**	**Posttest (24 h after training)**
**Quadriceps (^º^)**			
Active Recovery	15	130.1 ± 7.0^*^ (−0.29)	127.2 ± 6.7 (−0.42)
Passive Recovery	16	132.2 ± 7.3^*^	129.9 ± 6.2
**Hamstring (^º^)**			
Active Recovery	15	79.4 ± 7.1^#^ (−0.94)	79.3 ± 8.1 (−0.48)
Passive Recovery	16	86.2 ± 7.3	83.3 ± 8.0
**Adductor (^º^)**			
Active Recovery	15	41.2 ± 7.8 (−0.23)	40.1 ± 5.9 (−0.49)
Passive Recovery	16	42.7 ± 4.4	42.7 ± 4.4
**Gastrocnemius (^º^).**			
Active Recovery	15	26.6 ± 6.9 (0.27)	25.5 ± 4.3 (−0.15)
Passive Recovery	16	25.1 ± 3.4	26.1 ± 3.2

* Significant differences (p < 0.05) with posttest.

# Significant differences (p < 0.05) with passive recovery group.

ES (with respect to passive recovery group values) are shown in parentheses.

## References

[b1-jhk-31-121] Andersson H, Raastad T, Nilsson J, Paulsen G, Garthe I, Kadi F (2008). Neuromuscular fatigue and recovery in elite female soccer: effects of active recovery. Med Sci Sports Exerc.

[b2-jhk-31-121] Baldari C, Videira M, Madeira F, Sergio J, Guidetti L (2004). Lactate removal during active recovery related to the individual anaerobic and ventilatory thresholds in soccer players. Eur J Appl. Physiol.

[b3-jhk-31-121] Balsom PD, Ekblom B (1994). Evaluation of physical performance. Football (soccer).

[b4-jhk-31-121] Barnett A (2006). Using recovery modalities between training sessions in elite atheletes: does it help?. Sports Med.

[b5-jhk-31-121] Bompa T (1999). Periodization: Theory and methodology of training.

[b6-jhk-31-121] Borg G (1998). Borg’s perceived exertion and pain scales.

[b7-jhk-31-121] Dabedo B, White J, George KP (2004). A survey of flexibility training protocols and hamstrings strains in professional football clubs in England. Br J Sports Med.

[b8-jhk-31-121] Dawson B, Gow S, Modra S, Bishop D, Stewart G (2005). Effects of immediate post-game recovery procedures on muscle soreness, power and flexibility levels over the next 48 hours. J Sci Med Sport.

[b9-jhk-31-121] Dupont G, Nedelec M, McCall A, McCormack D, Berthoin S, Wisløff U (2010). Effect of 2 soccer matches in a week on physical performance and injury rate. Am J Sports Med.

[b10-jhk-31-121] Hoff J, Wisløff U, Engen LC, Kemi OJ, Helgerud J (2002). Soccer specific aerobic endurance training. Br J Sports Med.

[b11-jhk-31-121] Hopkins W, Batterham AM (2006). Making meaningful inferences about magnitudes. Int J Sports Physiol Perform.

[b12-jhk-31-121] Impellizzeri FM, Rampinini E, Coutts AJ, Sassi A, Marcora SM (2004). Use of RPE-based training load in soccer. Med Sci Sports Exerc.

[b13-jhk-31-121] King M, Duffield R (2009). The effects of recovery interventions on consecutive days of intermittent sprint exercise. J Strength Cond Res.

[b14-jhk-31-121] Kinugasa T, Kilding AE (2009). A comparison of post-match recovery strategies in youth soccer players. J Strength Cond Res.

[b15-jhk-31-121] Montgomery P, Pyne D, Hopkins W, Dorman J, Cook K, Minahan C (2008). The effect of recovery strategies on physical performance and cumulative fatigue in competitive basketball. J Sports Sci.

[b16-jhk-31-121] Odetoyinbo K, Wooster B, Lane A, Reilly T, Korkusuz F (2009). The effect of a succession of matches on the activity profiles of professional soccer players. Science and Football VI.

[b17-jhk-31-121] Olsen O, Myklebust G, Engebrestsen L, Holme I, Bahr R (2005). Exercises to prevent lower limb injuries in youth sports: cluster randomised controlled trial. Br Med J.

[b18-jhk-31-121] Reilly T, Drust B, Clarke N (2008). Muscle fatigue during football match-play. Sports Med.

[b19-jhk-31-121] Reilly T, Ekblom B (2005). The use of recovery methods post-exercise. J Sports Sci.

[b20-jhk-31-121] Reilly T, Rigby M, Sprinks W, Reilly T, Murphy A (2002). Effect on active warm-down following competitive soccer. Science and Football IV.

[b21-jhk-31-121] Rey E, Lago-Peñas C, Lago-Ballesteros J, Casáis L, Dellal A (2010). The effect of a congested fixture period on the activity of elite soccer players. Biol Sport.

[b22-jhk-31-121] Riach IE, McDonald R, Newell J (2004). Nutritional and anthropometric assessment of elite soccer players. J Sports Sci.

[b23-jhk-31-121] Robson-Ansley P, Gleeson M, Ansley L (2009). Fatigue management in the preparation of Olympic athletes. J Sports Sci.

[b24-jhk-31-121] Rodacki ALF, Fowler NE, Bennett SJ (2002). Vertical jump coordination: fatigue effects. Med Sci Sports Exerc.

[b25-jhk-31-121] Ryan ED, Herda TJ, Costa PB, Defreitas JM, Beck TW, Stout J, Cramer JT (2009). Determining the minimum number of passive stretches necessary to alter musculotendinous stiffness. J Sports Sci.

[b26-jhk-31-121] Shephard RJ (2002). Ethics in exercise science research. Sports Med.

[b27-jhk-31-121] Stølen T, Chamari K, Castagna C, Wisløff U (2005). Physiology of soccer. An update. Sports Med.

[b28-jhk-31-121] Tessitore A, Meeusen R, Cortis C, Capranica L (2007). Effects of different recovery interventions on anaerobic performances following preseason soccer training. J Strength Cond Res.

[b29-jhk-31-121] Witvrouw E, Danneels L, Asselman P, D’Have T, Cambier D (2003). Muscle flexibility as a risk factor developing muscle injuries in male professional soccer players. Am J Sports Med, 2003.

